# Adjunctive Minocycline in Acute Ischemic Stroke: A Systematic Review and Meta‐Analysis of Randomized Controlled Trials

**DOI:** 10.1002/brb3.71576

**Published:** 2026-07-09

**Authors:** Samaha Khalid, Malik Aon Ali Abbas, Muhammad Sohaib Shahid, Muhammad Nihal Moarij Azeem Malik, Mahnoor Javed, Abdur Rehman, Saad Ashraf, Muhammad Daoud Tariq, Deepak Rai

**Affiliations:** ^1^ Department of Internal Medicine Foundation University Medical College Islamabad Pakistan; ^2^ Medical College Aga Khan University Karachi Pakistan; ^3^ Department of ENT and Head and Neck Surgery Benazir Bhutto Hospital Rawalpindi Pakistan; ^4^ Department of Medicine Dow University of Health Sciences Karachi Pakistan; ^5^ National Academy of Medical Sciences Bir Hospital Kathmandu Nepal

**Keywords:** acute ischemic stroke, minocycline, neuroprotection, randomized controlled trials, recovery

## Abstract

**Background:**

Acute ischemic stroke (AIS) is a leading cause of mortality and long‐term disability worldwide. Despite advances in acute stroke management, many patients continue to experience substantial neurological disability and incomplete recovery. Minocycline, a tetracycline antibiotic with anti‐inflammatory and neuroprotective properties and good blood–brain barrier penetration, has been investigated as a potential adjunctive therapy in AIS.

**Methods:**

A systematic search of major databases was conducted identifying randomized controlled trials evaluating minocycline in patients with AIS. Outcomes included functional and neurological recovery assessed using the modified Rankin Scale (mRS), National Institutes of Health Stroke Scale (NIHSS), and Barthel index (BI), as well as mortality and vascular events. Pooled effect estimates were calculated using random‐effects meta‐analysis.

**Results:**

Seven RCTs comprising 2197 patients were included, with 1093 receiving minocycline and 1104 receiving placebo or standard care. Functional independence at 3 months (mRS 0–2) showed no significant difference between groups (RR 1.28, 95% CI 0.97–1.69; *I* ^2^ = 84.9%). Minocycline was associated with lower NIHSS scores (MD −2.45, 95% CI −4.32 to −0.59; *I* ^2^ = 88.6%) and higher BI scores (MD 12.52, 95% CI 6.28–18.76; *I* ^2^ = 47.0%). Mortality (RR 0.66, 95% CI 0.39–1.090 and stroke recurrence or MI showed no significant differences (RR 0.97, 95% CI 0.69–1.37).

**Conclusions:**

Although a consistent improvement in functional independence was not demonstrated, minocycline showed signals of benefit in selected neurological outcomes with a favorable safety profile. These findings support the need for adequately powered, multicenter randomized trials to clarify its therapeutic role.

## 1 Introduction

Acute ischemic stroke (AIS) is a medical emergency characterized by the sudden loss of brain function resulting from disruption of cerebral blood flow (Laurent et al. 2025). Given the rapid progression of neuronal injury following ischemia, early recognition and timely intervention are critical to minimize brain damage and prevent adverse outcomes (Mendelson and Prabhakaran 2021).

AIS is diagnosed clinically and confirmed with neuroimaging, most commonly non‐contrast computed tomography (CT), which is mainly used to exclude intracranial hemorrhage (Musuka et al. 2015). Current management includes evidence‐based acute interventions and supportive care aimed at reducing neurological injury and improving functional recovery (NICE 2022). Despite advancements in stroke treatment, many patients continue to experience substantial disability and incomplete neurological recovery. These ongoing challenges highlight the need for adjunctive therapeutic strategies that target secondary injury mechanisms and promote recovery after stroke.

Neuroprotective agents aim to mitigate secondary brain injury by targeting key mechanisms within the ischemic cascade, including excitotoxicity, oxidative stress, inflammation, and apoptosis (Vos et al. 2022). Among these, minocycline is of particular interest due to its high lipophilicity, which facilitates blood–brain barrier penetration, as well as its low cost and favorable safety profile (Fagan et al. 2011). These characteristics support its investigation as a potential adjunctive therapy in AIS.

Previous systematic reviews and meta‐analyses (Malhotra et al. 2018) evaluating minocycline in acute stroke have reported inconclusive and heterogeneous findings, with inconsistent results across randomized controlled trials, likely due to small sample sizes, variability in treatment protocols, and methodological limitations. These uncertainties, alongside calls for larger, well‐designed trials, have limited the reliability of prior conclusions.

Recently, the EMPHASIS trial, a large multicenter, double‐blind randomized controlled trial demonstrated a significant improvement in functional outcomes with minocycline without safety concerns, providing robust new evidence that may influence existing conclusions (Lu et al. 2026). Accordingly, an updated systematic review and meta‐analysis is warranted to incorporate this evidence and provide a more precise evaluation of its efficacy and safety.

## 2 Methods

### 2.1 Protocol Registration

This systematic review and meta‐analysis was conducted in accordance with the Preferred Reporting Items for Systematic Reviews and Meta‐Analyses (PRISMA) guidelines (Page et al. 2021).

The primary objective was to systematically evaluate the safety and efficacy of minocycline in management of AIS through quantitative synthesis of evidence from randomized controlled trials. The protocol was prospectively registered with PROSPERO (CRD420261321955).

### 2.2 Study Sources and Search Strategy

A comprehensive electronic search was carried out using a variety of databases, including Web of Science, PubMed, Embase, Google Scholar, Scopus, and Cochrane Central Register of Controlled Trials (CENTRAL), from inception to February 17, 2026. The search string utilized a combination of Medical Subject Headings (MeSH) and free‐text keywords including “Stroke,” “AIS,” “Ischemic stroke,” “Minocycline,” and “Cerebral infarction.” Boolean operators (AND, OR) were applied to maximize sensitivity. To ensure literature saturation, reference lists of identified trials and prior meta‐analyses were manually screened. Gray literature, including conference abstracts and trial registry records, was considered where relevant. Only studies published in English were included due to resource constraints for translation, which may have introduced language bias. The full search strategy for each database is archived in Table S1.

### 2.3 Eligibility Criteria

Eligibility criteria were predefined according to the Population, Intervention, Comparator, Outcomes, and Study designs (PICOS) framework to ensure structured and transparent study selection process. Randomized controlled trials evaluating minocycline as a therapeutic intervention in patients with AIS were considered eligible. Included studies were required to report at least one efficacy outcome or one safety outcome. Studies were excluded if they lacked comparator or focused exclusively on hemorrhagic stroke or were observational studies, reviews, editorials, or case reports. In addition, the studies, which did not provide sufficient data for quantitative synthesis or included mixed stroke populations without extractable data specific to AIS patients, were also excluded.

### 2.4 Study Selection

All records identified through the database search were imported into Rayyan AI, and detected duplicates were removed by the reviewers (Ouzzani et al. 2016) . Titles and abstracts were screened to identify potentially eligible studies, followed by full‐text assessment against the predefined eligibility criteria. Study selection was conducted independently by two reviewers (A.A.A and S.K.), from initial literature search to final inclusion of study, and disagreements were resolved through discussion and consensus with a third author (MSS). The study selection process was documented in accordance with PRISMA guidelines and presented in a PRISMA flow diagram.

### 2.5 Data Extraction

Data were extracted independently by two reviewers (A.A.A and S.K.) using a standardized form. Any disagreements were resolved through discussion and consensus, and where necessary a third reviewer was consulted (MSS). Extracted variables included study characteristics, participant demographics, intervention details (minocycline dosage and duration), comparator characteristics, reported outcomes, and follow‐up duration. Data were extracted on intention‐to‐treat (ITT) principles wherever possible. For dichotomous outcomes, event counts and total sample sizes were extracted. For continuous outcomes, means and standard deviations were extracted; where necessary, data were converted or estimated using standard methods.

### 2.6 Quality Assessment

Risk of bias in the included studies was independently assessed by two reviewers (MNMAM, MJ) using the Cochrane Risk of Bias 2 (RoB 2) tool (Sterne et al. 2019), which evaluates bias across five domains: the randomization process, deviations from intended interventions, missing outcome data, outcome measurement, and selection of reported results. Each study was classified as having low risk of bias, some concerns, or high risk of bias according to RoB 2 criteria. Discrepancies between reviewers were resolved through discussion and consensus. The certainty for evidence of each outcome was assessed using the GRADE approach, which incorporates risk of bias alongside inconsistency, indirectness, imprecision, and publication bias. A summary of findings table was generated to present the certainty of evidence for key outcomes.

### 2.7 Outcomes

The primary efficacy outcomes were functional and neurological recovery at 3 months, including functional independence measured by a modified Rankin Scale (mRS) score of 0–2, neurological deficit assessed using the National Institutes of Health Stroke Scale (NIHSS), and functional performance measured by the Barthel index (BI). A favorable functional outcome, defined as an mRS score of 0–1 at 3 months, was assessed as an additional outcome where reported. Exploratory analyses were conducted at earlier follow‐up intervals (5–7 days and 30 days) where data were available. Secondary safety outcomes included all‐cause mortality and recurrent stroke or myocardial infarction.

### 2.8 Statistical Analysis

Statistical analyses were performed using R software (version 2025.05.1 + 513) with the meta package (). For dichotomous outcomes, pooled effect estimates were calculated as risk ratios (RRs) with 95% confidence intervals (CIs) using the Mantel–Haenszel method, while mean differences (MDs) were used for continuous outcomes measured on the same scale. Meta‐analyses were conducted using a random‐effects model to account for potential clinical and methodological heterogeneity across studies, with between‐study variance (*τ* ^2^) estimated using the Paule–Mandel method.

Statistical heterogeneity was assessed using the Cochran Q test and quantified using the *I* ^2^ statistics, with values of 25%, 50%, and 75% representing low, moderate, and high heterogeneity, respectively (Higgins et al. 2003). When studies reported zero events in one treatment arm, a continuity correction of 0.5 was applied to enable effect size estimation.

Sensitivity analyses were performed using a leave‐one‐out approach to evaluate the influence of individual studies on pooled estimates. Baujat plots were generated to examine the contribution of individual studies to overall heterogeneity. Subgroup analysis was not performed due to limited number of included studies and insufficient data to allow meaningful comparisons. Publication bias was not formally assessed due to the limited number of included studies per outcome (< 10), as funnel plots and statistical tests for asymmetry are unreliable in such cases (Higgins et al. 2019) All statistical tests were two‐sided, and *p* value of < 0.05 was considered statistically significant.

## 3 Results

### 3.1 Study Selection and Characteristics

**FIGURE 1:**
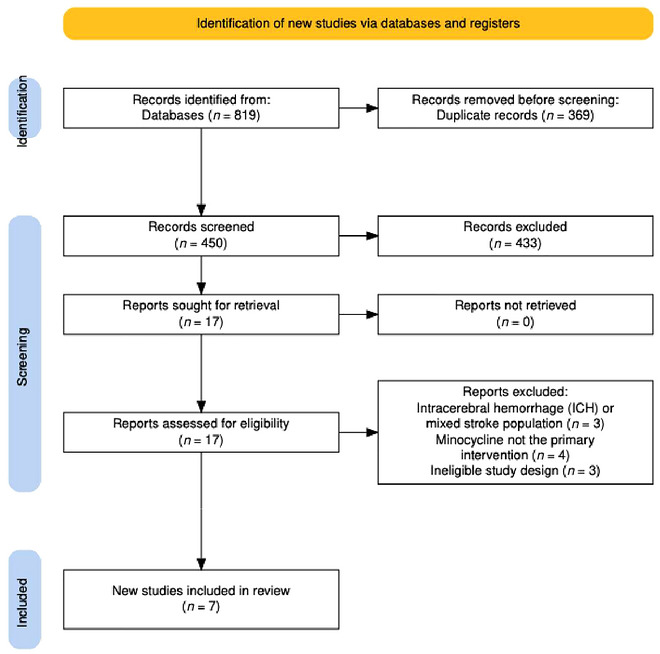
PRISMA flowchart.

The initial electronic search identified 1042 potentially relevant citations. Following the removal of duplicates and irrelevant records, including case reports, editorials, review articles, and wrong titles, 566 records were further screened. The records were further excluded as they lacked proper comparator or wrong population, and 17 records underwent full‐text evaluation. One record was removed as it lacked proper outcome (Switzer et al. 2011) Three of the records included patients with intracranial hemorrhage and hence were excluded (Kohler et al. 2013; Chang et al. 2017; Fouda et al. 2017). After careful evaluation, 7 randomized controlled trials were included which had relevant comparators and results (Lu et al. 2026; Lampl et al. 2007; Padma Srivastava et al. 2012); Blacker et al. 2013; Amiri‐Nikpour et al. 2015; Shamsaei and Mohammadi 2017; Singh et al. 2019). The PRISMA flow diagram detailing the attrition of studies is presented in Figure 1.

The total pooled cohort consisted of 2197 patients, of whom 1093 were on minocycline therapy in addition to standard stroke management compared with 1104 on placebo for acute ischemicstroke. Baseline demographics and clinical characteristics were generally comparable between the treatment and control groups across the included studies, including age and the prevalence of common vascular risk factors such as hypertension, diabetes mellitus, and dyslipidemia. Comprehensive study characteristics and patient‐level baseline data are provided in Tables 1 and 2.

**TABLE 1:** Baseline characteristics of studies included. Comparison of minocycline versus placebo in acute ischemic stroke (AIS).

Study	Year	Origin	No. of patients		Minocycline dose	Route, duration of treatment	Minocycline administration from symptoms onset, hours	Outcomes
			Minocycline	Placebo				
Amiri‐Nikpour et al.	2015	Iran	26	27	200 mg, once daily	Oral, 5 days	6–24 h	NIHSS
Blacker et al.	2013	Australia	21	23	200 mg, twice daily	IV, 5 doses	< 6 h	NIHSS, mRS, BI
Lampl et al.	2007	Israel	74	77	200 mg, once daily	Oral, 5 days	6–24 h	NIHSS, mRS, BI
Lu et al.	2026	China	862	862	200 mg, twice daily	Oral, 4 days	72 h	NIHSS, mRS, BI
Padma Srivastava et al.	2012	India	23	27	200 mg, once daily	Oral, 5 days	6–24 h	NIHSS, mRS, BI
Shamsaei et al.	2017	Iran	18	18	200 mg, once daily	Oral, 5 days	6–24 h	NIHSS
Singh et al.	2019	Singapore	69	70	200 mg, once daily	Oral, 5 days	3–48 h	mRS

Abbreviations: BI, Barthel index; IV, intravenous; mRS, modified Rankin Scale; NIHSS, National Institutes of Health Stroke Scale.

**TABLE 2:** Baseline characteristics of patients included. Patient demographics and clinical characteristics by treatment group.

Trial	Age (years) Mean ± SD Mino, Placebo	Males (%) Mino, Placebo	HTN (%) Mino, Placebo	DM (%) Mino, Placebo	HLD (%) Mino, Placebo	Smoker (%) Mino, Placebo	Prior AIS/TIA (%) Mino, Placebo	Prior heart disease (%) Mino, Placebo
Amiri‐Nikpour et al.	65.23 ± 9, 66.52 ± 7.8	46.15, 48.14	69.23, 77.78	17.39, 29.62	46.15, 40.74	34.61, 37.03	19.23, 18.51	34.61, 29.62
Blacker et al.	68.26 ± 14.19, 68 ± 12.95	65.21, 60.86	47.82, 65.21	13.04, 30.43	52.17, 52.17	47.82, 47.82	13.04, 4.34	21.73/ 21.73
Lu et al.	64.67 ± 11.1, 64.3 ± 10.4	66.5, 67.1	64.3, 66.4	33.2, 32.5	43.9, 42.1	37.7, 41.6	27.6, 29.9	11, 13.2
Lampl et al.	67.2 ± 11.1, 66.2 ± 11.1	63.51, 66.23	60.81, 64.93	36.36, 25	24.32, 35.06	27.02, 31.16	22.97, 22.07	29.72, 31.16
Srivastava et al.	52.7 ± 15.3, 57 ± 14.2	56.52, 66.67	65.21, 70.37	17.39, 29.62	43.47, 44.44	30.43, 13.81	NR	26.08, 37.03
Shamsaei et al.	69 ± 10.3, 68 ± 10.3	44.44, 38.89	50, 55.56	50, 16.67	27.8, 33.33	33.33, 22.22	NR	NR
Singh et al.	61.5 ± 14.8, 62.6 ± 11.1	72.5, 74.3	73.9, 75.7	29.1, 38.5	39.2, 52.8	24.6, 35.7	4.4, 2.9	14.5, 7.2

Abbreviations: AIS, acute ischaemic stroke; DM, diabetes mellitus; HLD, hyperlipidaemia; HTN, hypertension; Mino, minocycline; TIA, transient ischemic attack.

### 3.2 Risk of Bias

Risk of bias was assessed using the Cochrane RoB 2 tool. Overall, two trials were judged to be at low risk of bias, three had some concerns, and two were classified as high risk of bias. High risk of bias was primarily driven by deviations from intended interventions and concerns in outcome measurement. Detailed domain‐level assessments are provided in the summary (Figure S1).

### 3.3 Efficacy Outcomes

#### 3.3.1 Modified Rankin Scale

**FIGURE 2:**
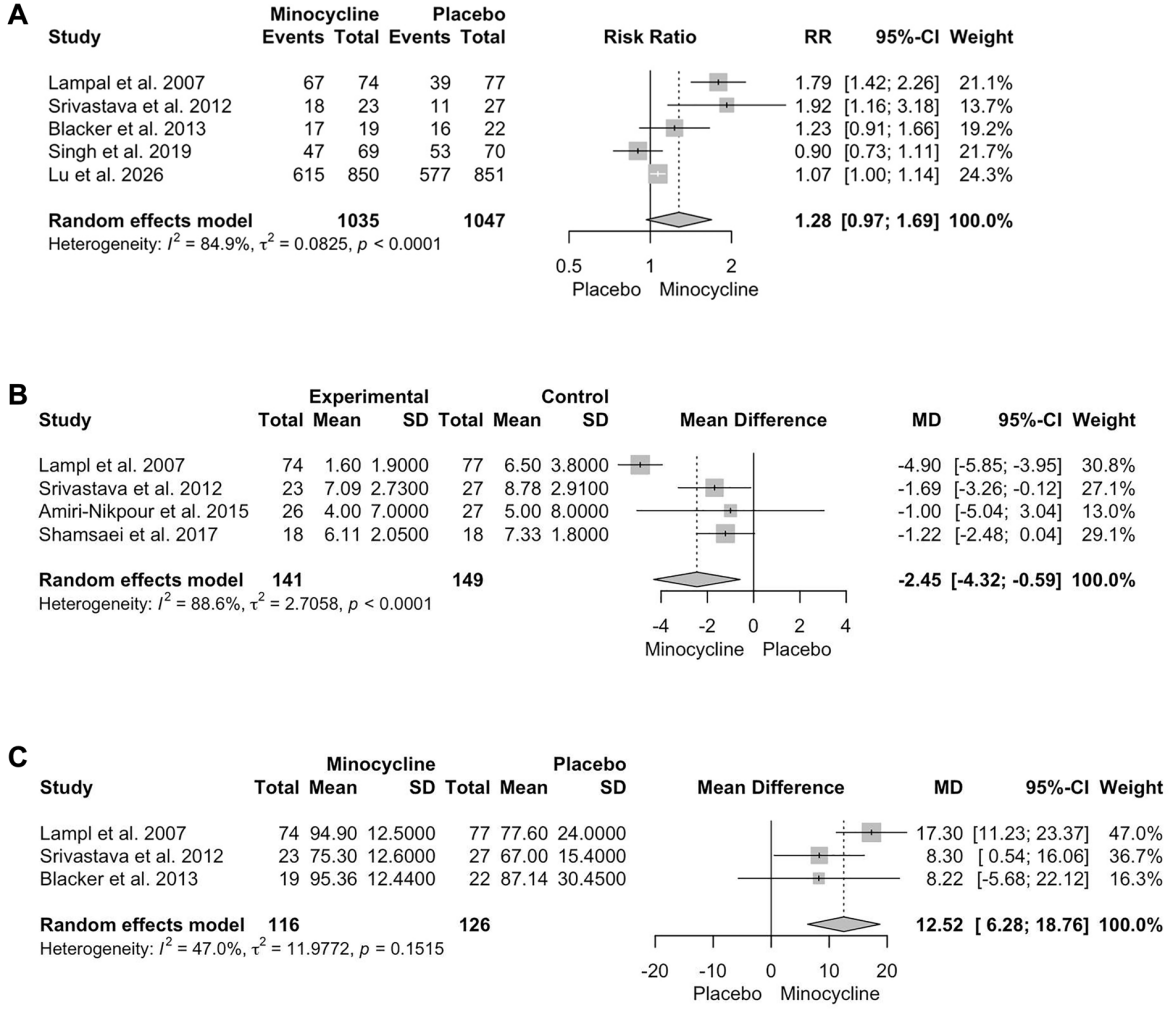
Forest plots illustrating the effects of minocycline in patients with acute ischemic stroke. Individual study estimates and pooled effects are presented with 95% confidence intervals (CIs) using a random‐effects model. Squares represent individual study estimates (size proportional to study weight), horizontal lines indicate 95% CIs, and the diamond represents the pooled effect estimate. (A) Forest plot showing the association of minocycline (compared to placebo) with 3‐month favorable functional outcome defined as mRS scores of 0–2. (B) Forest plot showing the association of minocycline (compared to placebo) with 90‐day NIHSS score. (C) Forest plot showing the pooled effect of minocycline on functional independence measured by the Barthel index (BI) in patients with acute ischemic stroke.

The pooled random‐effects estimate for mRS (0–2) at 3 months was derived from five RCTs including 2082 participants (RR: 1.28, 95% CI 0.97–1.69) (Figure 2A). There was no statistically significant difference between groups. Substantial heterogeneity was observed across studies *I^2^* = 84.9% after excluding Lampl 2007 (Lampl et al. 2007), it dropped to *I^2^* = 65.4% (Table S2). Exploratory analyses at earlier follow‐up at 30 days showed comparable patterns with no significant differences observed (MD: −0.76; 95% CI: −1.65 to 0.13; *I* ^2^ = 89.9%) (Figure S2).

A favorable functional outcome at 3 months (defined as mRS score 0–1) was reported in five RCTs (RR: 1.39, 95% CI 0.73–2.67), indicating no statistically significant difference between groups (Figure S3).

#### 3.3.2 NIHSS Score

The pooled random‐effects estimate for NIHSS at 3 months was derived from four RCTs including 290 participants (MD = −2.45, 95% CI −4.32 to −0.59), indicating lower NIHSS score in the minocycline group (Figure 2B). Substantial heterogeneity was observed (*I* ^2^ = 88.6%) after excluding Lampl 2007 (Lampl et al. 2007) *I* ^2^ = 0 (Table S2). Exploratory analyses at earlier follow‐up intervals showed no significant differences at 5–7 days (MD: −0.78; 95% CI: −1.62 to 0.06; *I* ^2^ = 44.4%) (Figure S4) and 30 days (MD: −2.28; 95% CI: −5.75 to 1.19; *I* ^2^ = 91.0%) (Figure S5).

#### 3.3.3 BI Score

The pooled random‐effects estimate for BI at 3 months was derived from three RCTs including 242 participants (MD = 12.52, 95% CI 6.28–18.76; *p* = < 0.0001), indicating significantly higher BI scores in the minocycline group (Figure 2C). Moderate heterogeneity was observed (*I* ^2^ = 47.0%) after excluding Lampl 2007 (Lampl et al. 2007) *I* ^2^ *= 0* (Table S2). Exploratory analyses at earlier follow‐up intervals showed no statistically significant difference with pooled estimates at 5–7 days (MD: 13.47; 95% CI: −7.49 to 34.44; *I* ^2^ = 90.1%) (Figure S6) and 30 days (MD: 11.49; 95% CI: −1.13 to 24.12; *I* ^2^ = 82.5%) (Figure S7).

### 3.4 Safety Outcomes

#### 3.4.1 Mortality

Mortality was reported in four RCTs, including 2036 participants. The pooled random‐effects estimate showed no statistically significant difference between groups (RR: 0.66; 95% CI: 0.39–1.09), with no heterogeneity observed across studies (Figure S8). Although the point estimate was lower in the minocycline group, the CI was wide and included the possibility of no effect.

#### 3.4.2 Stroke Recurrence or Myocardial Infarction

Stroke recurrence or myocardial infarction was reported in four randomized controlled trials including 2040 participants. The pooled random‐effects estimate was (RR: 0.97; 95% CI 0.69–1.37), with no heterogeneity observed across studies (*I* ^2^ = 0%) (Figure S9).

The certainty of evidence for key outcomes, assessed using the GRADE approach, is summarized in the Summary of Findings table (Table 3). Overall, the certainty of evidence was low for functional and neurological outcomes, including mRS (0–2), NIHSS, and BI, primarily due to substantial heterogeneity and imprecision. In contrast, the certainty of evidence was moderate for mortality and recurrent vascular events, although these outcomes showed no significant difference between groups.

**TABLE 3:** Summary of findings.

**Certainty assessment**							**№ of patients**		**Effect**		**Certainty**	**Importance**
**№ of studies**	**Study design**	**Risk of bias**	**Inconsistency**	**Indirectness**	**Imprecision**	**Other considerations**	**Minocycline**	**Placebo**	**Relative (95% CI)**	**Absolute (95% CI)**		
**Functional independence; mRS (score 0–2) (follow‐up: mean 3 months)**												
5	Randomized trials	Serious^a^	Serious^b^	Not serious	Not serious	None	764/1035 (73.8%)	696/1047 (66.5%)	**RR 1.28** (0.97–1.69)	**186 more per 1000** (from 20 fewer to 459 more)	⨁⨁◯◯ Low^a,b^	CRITICAL
**NIHSS score (follow‐up: mean 3 months)**												
4	Randomized trials	Not serious	Serious^b^	Not serious	Serious^c^	None	141	149	—	MD **2.45 lower** (4.32 lower to 0.59 lower)	⨁⨁◯◯ Low^b,c^	CRITICAL
**Barthel index (follow‐up: mean 3 months)**												
3	Randomized trials	Serious^a^	Not serious	Not serious	Serious^c^	None	116	126	—	MD **12.52 higher** (6.28 higher to 18.76 higher)	⨁⨁◯◯ Low^a,c^	IMPORTANT
**Mortality (follow‐up: mean 3 months)**												
4	Randomized trials	Not serious	Not serious	Not serious	Serious^c^	None	22/1018 (2.2%)	34/1018 (3.3%)	**RR 0.66** (0.39–1.09)	**11 fewer per 1000** (from 20 fewer to 3 more)	⨁⨁⨁◯ Moderate^c^	CRITICAL
**Recurrent stroke or myocardial infarction (follow‐up: mean 3 months)**												
4	Randomized trials	Not serious	Not serious	Not serious	Serious^c^	None	58/1019 (5.7%)	60/1021 (5.9%)	**RR 0.97** (0.69–1.37)	**2 fewer per 1000** (from 18 fewer to 22 more)	⨁⨁⨁◯ Moderate^c^	CRITICAL

Abbreviations: CI, confidence interval; MD, mean difference; RR, risk ratio.

^a^Downgraded for risk of bias due to high risk in ≥ 2 studies, particularly related to outcome assessment.

^b^Substantial statistical heterogeneity was observed (*I* ^2^ > 75%).

^c^Downgraded for imprecision due to limited sample size and wide confidence intervals.

Influence diagnostics were explored using Baujat plots (Figure S10). For the mRS outcome, Lu et al. (2026) contributed most to heterogeneity, while Lampl et al. (2007) had the greatest influence on the pooled estimate (Lu et al. 2026; Lampl et al. 2007). For NIHSS and BI outcomes, Lampl et al. (2007) contributed most to both heterogeneity and influence (Lampl et al. 2007).

Certainty of evidence was moderate for major clinical outcomes but lower for others due to imprecision and inconsistency (Table 3).

## 4 Discussion

In this systematic review of randomized controlled trials, minocycline in AIS showed a mixed but overall encouraging pattern of benefit at 3 months. Pooled analysis of the most commonly used functional outcome, mRS (score of 0–2), did not show a statistically significant benefit compared with placebo (RR 1.28, 95% CI 0.97–1.69). Improvements were observed in neurological deficit and functional performance, with lower NIHSS scores (MD −2.45, 95% CI −4.32 to −0.59) and higher BI scores (MD 12.52, 95% CI 6.28–18.76). The magnitude of these effects is within ranges that have been associated with clinically relevant differences in stroke outcomes in prior literature. In particular, small changes in NIHSS (e.g., ≥ 2‐point differences) have been linked to meaningful differences in functional outcomes and prognosis, while changes in the BI exceeding minimal clinically important difference thresholds and approaching larger increments (e.g., around 10 points) have been used to reflect more substantial functional recovery (Siegler et al. 2013; Hsieh et al. 2007). However, these findings were derived from a limited number of smaller trials with substantial heterogeneity and should therefore be interpreted with caution.

The discordance between outcomes warrants careful interpretation. Although minocycline was associated with improved NIHSS and BI scores at 3 months, this benefit was not mirrored by a statistically significant increase in primary mRS (score of 0–2) outcome. This may reflect differences in the way these measures capture recovery. Continuous scales such as NIHSS and BI may detect modest improvements that are not sufficient to shift patients across an mRS threshold. Importantly, the positive NIHSS and BI findings were derived from fewer and substantially smaller trials, resulting in less precise estimates with greater uncertainty, whereas the mRS analysis incorporated a larger evidence base, including the most adequately powered studies. As such, the mRS findings likely provide a more robust and reliable estimate of overall functional outcome.

Our findings are partly consistent with the prior meta‐analysis by Malhotra et al. (Malhotra et al. 2018), which reported significant benefit of minocycline in the AIS subgroup for functional independence (defined as mRS 0–2), as well as improvements in BI and NIHSS at 3 months. However, unlike their AIS subgroup analysis, our pooled mRS analysis did not show a statistically significant improvement in functional independence (mRS 0–2). It is important to distinguish this from more stringent definitions of favorable functional outcome (mRS 0–1), which reflects an excellent functional outcome with minimal or no disability. This suggests that while minocycline may improve selected recovery measures after AIS, its effect on overall functional independence remains less certain in the updated ischemic stroke evidence base.

The biological rationale for minocycline in AIS is supported by the understanding that ischemic injury extends beyond the initial loss of perfusion into a secondary phase characterized by neuroinflammation, microglial activation, blood–brain barrier disruption, and matrix metalloproteinase‐mediated injury (Lakhan et al. 2009; del Zoppo et al. 2007; Candelario‐Jalil et al. 2022). Preclinical and translational studies suggest that minocycline may attenuate several of these pathways by reducing pro‐inflammatory microglial signaling and cytokine expression, inhibiting MMP‐2 and MMP‐9 activity, limiting blood–brain barrier injury, and modulating apoptotic pathways through effects on cytochrome c release and caspase activation (Candelario‐Jalil et al. 2022; Wang et al. 2003; Naderi et al. 2020; Tao et al. 2013). These mechanisms provide a biologically plausible explanation for the observed improvements in neurological deficit and functional performance, as reflected by NIHSS and BI outcomes. Microglial responses after ischemic stroke are temporally heterogeneous, such that the commonly used M1/M2 classification is best understood as an oversimplified heuristic rather than a strict biological dichotomy (Zhao et al. 2017). Nevertheless, experimental data suggest that minocycline may promote a shift toward a more anti‐inflammatory (M2‐like) microglial state while attenuating (M1‐like) pro‐inflammatory activation (Lu et al. 2021).

From a clinical perspective, the present findings support the potential role of minocycline as an adjunctive therapy in AIS, warranting further investigation. The overall pattern of improvement across neurologic and functional outcomes suggests that targeting post‐stroke inflammatory and secondary injury pathways may translate into measurable recovery benefits at 3 months. In this context, minocycline is of particular interest given its favorable safety profile, good tolerability at standard doses, and high lipophilicity, enabling effective blood–brain barrier penetration (Nazarian and Akhondi 2023;Minocycline: MedlinePlus Drug Information 2026); Saivin and Houin 1988). The overall findings suggest that the effect of minocycline may be context‐dependent; however, the absence of stratified analyses precludes conclusions regarding the influence of factors such as timing of administration and baseline stroke severity.

Sex differences in AIS are important, as men and women may differ in risk profile, stroke severity, recovery, and functional outcomes (Appelros et al. 2009). For minocycline, it is relevant given its apoptosis and blood‐brain barrier‐modulating mechanism (Fagan et al. 2011). In this review sex distribution was reported in baselines across included trials, but sex‐stratified outcomes were largely unavailable. One small trial suggested significant neurological improvement among males, but this finding was limited by small sample size and baseline imbalance (Amiri‐Nikpour et al. 2015). Conversely, the large EMPHASIS trial did not demonstrate a differential treatment effect by sex (Lu et al. 2026). Therefore, current evidence does not confirm sex specific effect of minocycline in AIS.

From a global health perspective, these findings are particularly relevant in low‐ and middle‐income countries, where access to timely and equitable stroke care remains limited due to disparities in healthcare infrastructure, resource availability, and organization of stroke services (Pandian et al. 2020; Mendis 2010). In this context, minocycline is of particular interest as a low‐cost, widely available agent with a favorable safety profile that can be administered without the need for specialized infrastructure (Qurashi et al. 2017). These considerations highlight the importance of evaluating therapies not only for efficacy but also for feasibility and impact in resource‐constrained healthcare settings.

Several limitations should be acknowledged when interpreting these findings. Most importantly, substantial heterogeneity was present across key pooled analyses. This heterogeneity was clinically plausible and likely reflects important differences between trials regarding route of administration and timing of minocycline initiation, background stroke care, and variation in the proportion of patients receiving thrombolysis or thrombectomy. Treatment initiation ranged from within 6 h to beyond 48 h, and differences in treatment protocols may have influenced the observed treatment effects.

Lampl et al. (2007) was a notable contributor to heterogeneity, as demonstrated by sensitivity analyses showing marked reductions in heterogeneity following its exclusion. In comparison to more recent trials this study was conducted in an earlier era of stroke care, employed an open‐label evaluator blinded design, enrolled a relatively small sample, and reported substantially larger treatment effects across NIHSS, mRS, and BI outcomes than those observed in subsequent studies. In addition, baseline imbalances in selected patient characteristics and medication use were reported between treatment groups. These methodological and clinical differences may have contributed to the variability observed across pooled analyses. Although timing of administration may be an important modifier of treatment effect, subgroup analyses based on early versus late initiation (e.g., ≤ 24 h vs. > 24 h) were not performed due to limited data and variability in reporting across studies. Most included trials did not report outcomes stratified according to stroke severity categories, precluding meaningful quantitative subgroup analyses.

In addition, the included studies were conducted across different eras of stroke care, which may limit comparability given the advent of mechanical thrombectomy. Risk of bias assessment using the Cochrane RoB 2 tool demonstrated that several included studies had a high overall risk of bias, particularly due to deviations from intended interventions and lack of blinding. Subgroup analysis by stroke severity was limited because trials did not report outcomes across NIHSS severity strata; therefore, pooled subgroup analysis by baseline NIHSS was not feasible. Sex‐specific interpretation was also limited, as most included trials did not provide sufficient sex‐stratified data for formal analysis; therefore, inference regarding differential effect by sex remains exploratory. Finally, mechanistic interpretation remains limited because biomarker and radiological data were sparse and showed no clear difference when present. Taken together, these factors temper the certainty of the pooled estimates and suggest that the observed signal of benefit should be interpreted with appropriate caution.

Given these limitations, future research should place greater emphasis on mechanistic and translational endpoints, as current clinical data provide insufficient insight into how minocycline exerts its effects in human stroke populations. Incorporation of imaging biomarkers, such as infarct volume and cerebral edema, alongside blood biomarkers including S100β, neurofilament light chain, and brain‐derived tau, would improve mechanistic understanding. Studies should also assess if minocycline efficacy and safety vary according to baseline‐NIHSS severity. Future trials should prospectively report sex‐stratified efficacy and safety outcomes to determine whether effect of minocycline differs between male and female patients. Consistent reporting of NIHSS and BI at 3 months may further enable more granular assessment of recovery.

## 5 Conclusion

Overall, current evidence does not demonstrate a consistent improvement in functional independence with minocycline in AIS, although signals of benefit are observed in selected measures of neurological and functional recovery. Confidence in these findings remains limited. Future research should focus on adequately powered, multicenter randomized controlled trials with standardized intervention protocols, including consistent dosing, timing, duration of therapy, and sex‐specific treatment effects to better define its therapeutic role. In addition, comparative studies against contemporary standards of stroke care are warranted, as many included trials predate widespread use of advanced reperfusion therapies, and the relevance of these findings to current practice remains to be clarified.

## Author Contributions

**Abdur Rehman**: writing – review and editing, supervision. **Samaha Khalid**: conceptualization, data curation, writing – original draft, writing – review and editing. **Muhammad Sohaib Shahid**: writing – original draft, writing – review and editing. **Deepak Rai**: writing – review and editing, supervision. **Malik Aon Ali Abbas**: conceptualization, formal analysis, writing – original draft, writing – review and editing. **Muhammad Nihal Moarij Azeem Malik**: software, writing – original draft, writing – review and editing. **Muhammad Daoud Tariq**: writing – review and editing, supervision. **Mahnoor Javed**: writing – original draft, writing – review and editing. **Saad Ashraf**: writing – review and editing, supervision.

## Ethics Statement

Ethical approval was not required for this study because it is a systematic review and meta‐analysis based on previously published data.

## Funding

The authors have nothing to report.

## Conflicts of Interest

The authors declare no conflicts of interest.

## Supporting information




**Supplementary Material**: brb371576‐sup‐0001‐SuppMat.docx

## Data Availability

The data that support the findings of this study are available within the article and its Supporting Information.
